# Influencing factors of clinical efficacy of roxadustat among hemodialysis patients

**DOI:** 10.1080/0886022X.2024.2308701

**Published:** 2024-02-12

**Authors:** Wenhui Wu, Nan Hu, Xiurong Li, Jia Di, Hua Zhou, Hongyan Niu, Min Yang

**Affiliations:** a Department of Nephrology, The Third Affiliated Hospital of Soochow University, Changzhou, China; b Department of Pharmacy, The Third Affiliated Hospital of Soochow University, Changzhou, China

**Keywords:** Roxadustat, renal anemia, blood trough concentration of roxadustat, clinical efficacy

## Abstract

**Objective:**

To explore independent influencing factors for clinical efficacy of roxadustat in hemodialysis patients.

**Methods:**

Hemodialysis patients treated with roxadustat were enrolled. The plasma trough concentrations of roxadustat were measured using liquid chromatography–tandem mass spectrometry (LC–MS/MS). A multiple logistic regression model was established to determine the factors that affect clinical efficacy of roxadustat in patients undergoing hemodialysis.

**Results:**

A total of 67 hemodialysis patients were enrolled in the study. The results showed that age, blood trough concentration of roxadustat, and baseline hemoglobin (Hb) level were independent factors of clinical efficacy of roxadustat (OR = 1.06, *p* = .025 for age; OR = 1.001, *p* = .037 for plasma concentration; and OR = 0.941, *p* = .003 for baseline Hb), with an AUC score of 0.859.

**Conclusions:**

Age, blood trough concentration of roxadustat, and baseline Hb level were independent influencing factors of the response to roxadustat in hemodialysis patients.

## Introduction

Anemia, a prevalent and serious complication associated with chronic kidney disease (CKD), may increase the risk of hospitalization and mortality, particularly in end-stage renal disease (ESRD) patients undergoing hemodialysis [1]. Renal anemia is caused by many factors such as reduced production of erythropoietin (EPO) in the kidneys, reduced red blood cell (RBC) survival, bleeding due to dysfunctional platelets, and profound iron deficiency due to renal failure [2]. The traditional treatment of renal anemia includes iron agents, parenteral erythropoiesis-stimulating agents (ESAs), and RBC transfusion [3]. However, the long-term use of iron agents is poorly tolerated and may cause several side effects. Multiple studies have shown that long-term use of ESAs may be associated with an increased risk of death and cardiovascular disease [4,5]. Moreover, some patients are hypo-response to ESAs [6,7]. This hyporesponsiveness may be induced by several factors including inflammation, nutritional status, and dialysis adequacy. Patients with hyporesponsiveness require a larger dose of ESAs, which increases the risk of morbidity and death invariably [8,9]. Therefore, new strategies are needed to reduce these side effects.

Roxadustat, orally administered, is a small molecule (molecular weight 352.34 g/mol) hypoxia-inducible factor (HIF) prolyl hydroxylase inhibitor that increases HIF transcriptional activity by stabilizing HIF-α subunits. Increased HIF transcriptional activity promotes erythropoiesis by increasing endogenous EPO levels. Furthermore, roxadustat can decrease hepcidin levels to improve iron availability, which is necessary for erythropoiesis [10]. Multiple studies have demonstrated that roxadustat had favorable or comparable efficacy for the treatment of anemia in CKD patients on dialysis or not on dialysis compared to ESAs [11–14].

The pharmacokinetics (PK) of roxadustat have been well characterized. It can be readily absorbed (1–2 h) after oral administration and is eliminated by metabolism. The apparent volume of distribution of roxadustat was 22–56 L, the average elimination half-life (*t*_1/2_) was 12 h (10.9–13.1 h), the plasma binding was 99%, and the PK were unaffected by both hemodialysis and peritoneal dialysis. Among hemodialysis patients, only 2.34 ± 1.26% of roxadustat is eliminated during hemodialysis [15]. The effect of roxadustat on EPO was observed 8 h after oral administration. However, the effect on hemoglobin (Hb) is observed after several weeks (two weeks or more), exhibiting a nearly linear, steady rise, and sustained persistence [15]. Multiple trials have demonstrated that roxadustat is a safe and effective drug for the treatment of renal anemia; however, little is known about the factors influencing the curative effect of roxadustat in patients with renal anemia. This study aimed to explore the independent factors that affect the curative effect of roxadustat in hemodialysis patients with renal anemia.

## Materials and methods

### Participants

This prospective clinical study was conducted at the Department of Blood Purification Center of Changzhou First People Hospital from 2020 to 2021. The inclusion criteria were as follows: age 18–90 years; patients receiving maintenance hemodialysis for more than 3 months; patients with roxadustat for more than 3 months; and willingness to participate in the study. The exclusion criteria were as follows: (a) patients with poor control of blood pressure; (b) patients with a history of malignancy; (c) patients with severe liver impairment or active hepatitis; (d) patients with hereditary hematologic diseases such as thalassemia, sickle cell anemia, or other known causes of anemia other than CKD; (e) patients with chronic infections, acute infections or active inflammatory diseases.

### Study design

**Figure 1.**
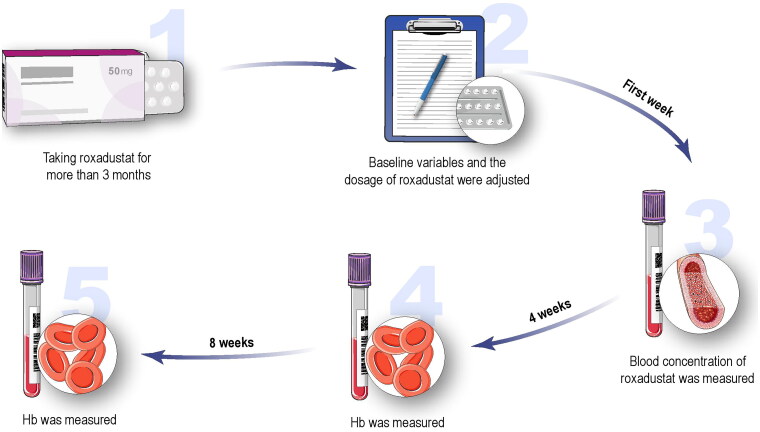
The flowchart of study design.

A total of 67 hemodialysis patients with renal anemia treated with roxadustat were included in the analysis. The drug was taken orally three times per week. The flowchart is shown in Figure 1. In the first week, the Hb level was tested, and the dosage of roxadustat was adjusted according to the change in Hb over the past weeks, as described in the instructions for roxadustat. After one week, the blood concentration of roxadustat was measured for a stable concentration. Hb levels were measured every 4 weeks for 8 weeks. The dosage of roxadustat was not adjusted within 8 weeks. Other study variables including age, sex, weight (kg), height (cm), body mass index (BMI, kg/m^2^), cause of kidney disease, serum creatinine (Scr, mg/dl), RBC (g/L), hematocrit (Hct, L/L), white blood cell count (×10^9^/L), neutrophils (×10^9^/L), platelets (×10^9^/L), lymphocytes (×10^9^/L), neutrophil-to-lymphocyte ratio (NLR), platelet-to-lymphocyte ratio (PLR), dosage of roxadustat (mg), baseline Hb (g/L), oral iron (ferrous succinate tablets, 100 mg per tablet), Hb after 4 weeks (g/L), Hb after 8 weeks (g/L), parathyroid hormone (PTH, pg/mL), serum ferritin (SF, ng/mL), previous anemia treatment from 3 to 6 months before enrollment and the single pool *Kt*/*V* (*spKt*/*V*) (*K*, dialyzer urea clearance; *t*, total dialysis session; *V*, volume of distribution of urea) were collected and measured in the first week. An *spKt*/*V* ration was calculated using the values after one HD session: *spKt*/*V* Daugirdas = –ln ([BUN_Post_/BUN_Pre_] – [0.008 × hour]) + ([4 – (3.5 × BUN_Post_/BUN_Pre_)] × UF_Vol_/weight). Previous anemia treatment included three types: recombinant human erythropoietin (rhEPO) treatment (3SBIO, S19980074), roxadustat treatment, and rhEPO conversion to roxadustat treatment. All patients were prescribed a dosage of one ferrous succinate tablet three times a day.

The study protocol was approved and supervised by the Ethics Committee of Changzhou First Hospital (no. 2023.041) and conducted in accordance with the ethical principles stated in the Declaration of Helsinki and applicable laws and regulations. Each participant was followed for 8 weeks.

### Method of blood trough concentration detection

Venous blood samples were collected before the oral administration of roxadustat in heparin tubes and centrifuged at 4000 r/min for 10 min to obtain plasma samples for analysis. Plasma trough concentrations of roxadustat were measured using liquid chromatography–tandem mass spectrometry [16]. Mycophenolic acid (MPA) was used as an internal standard. The gradient elution was performed on a Phenomenex Kinetex C18 column (3.0 mm × 100.0 mm, 2.6 μm) at a flow rate of 0.50 mL/minutes with mobile phase methanol (0.1% formic acid)–water (0.1% formic acid, 10 mmol/L ammonium acetate) solution. The injection volume was 5 μL. An electron spray ionization ion source was used for mass spectrometry and positive ion multiple reaction monitoring scanning. The quantification limit of roxadustat was 1 ng/mL. The intra- and inter-day RSD of roxadustat were <10%. The extraction recovery rate was 87.96–102.82%.

### Evaluation of clinical efficacy

Because the participants in this study were treated with roxadustat for more than 1 month, the dosage of roxadustat was reduced, which led to a weak fluctuation in Hb. Therefore, the response group was identified as a change in Hb from baseline ≥5 g/L, and the non-response group as <5 g/L.

### Statistical analysis

Data are expressed as the mean ± standard deviation and frequency or percentage for continuous and categorical variables, respectively. Both independent two-sample *t*-test analyses (for continuous variables) and Chi-square test (for categorical variables) were used to calculate the differences between groups. Multiple logistic regression analysis was performed to determine the factors influencing clinical efficacy. The predictive power of the model was evaluated using area under the receiver operating characteristic (AUROC) curve analysis, and the goodness-of-fit was evaluated using the Hosmer–Lemeshow statistical method. All statistical analyses were performed using SPSS version 26 (IBM, Armonk, NY).

## Results

### Baseline characteristics of the study population

The demographic characteristics and other covariates of the participants are listed in Table 1. A total of 67 hemodialysis patients were included in this observational study, of whom 67% were male and 33% were female, with an average age of 53.63 ± 13.02 years old. The average blood trough concentration of roxadustat was 521.85 ± 728.71 ng/mL (range of 5.23–4790 ng/mL) and the average dosage of roxadustat was 300 (210, 300) mg per week. The mean baseline Hb was 103.24 g/L. Mean ferritin level was 102.29 ng/mL and 46% of the patients were receiving oral iron therapy.

**Table 1.** Clinical characteristics of the study population and comparison of characteristics between the two groups.

Clinical characteristics	Total	Nonresponse (*n* = 38)	Response (*n* = 29)	*p*
Male (%)	67%	67%	68%	.918
Age (years)	53.63 ± 13.02	50.49 ± 11.43	58 ± 14.01	.019
Weight (kg)	61.86 ± 12.89	63.86 ± 15.09	59.23 ± 8.83	.122
Height (cm)	166.16 ± 7.47	166.34 ± 7.78	165.93 ± 7.17	.825
BMI (kg/m^2^)	22.29 ± 3.78	22.93 ± 4.45	21.46 ± 2.51	.091
Cause of kidney disease (%)				
Chronic glomerulonephritis	65.67%	74.36%	53.57%	.091
Diabetic nephropathy	22.39%	15.38%	32.14%	
Polycystic kidney	2.99%	0.00%	7.14%	
IgA nephropathy	2.99%	2.56%	3.57%	
Benign arteriolar nephrosclerosis	4.48%	7.69%	0.00%	
ANCA-associated nephritis	1.49%	0.00%	3.57%	
Duration of dialysis (months)	28.87 ± 34.86	30 ± 33.55	27.29 ± 37.18	.756
Previous treatment				.257
rhEPO	3%	2.6%	3.6%	
rhEPO to roxadustat	13.4%	10.3%	17.9%	
Roxadustat	80.6%	87.2%	71.4%	
None	3%	0%	7.1%	
*spKt*/*V*	1.36 ± 0.34	1.36 ± 0.36	1.37 ± 0.31	.965
Red blood cell (g/L)	3.52 ± 0.6	3.56 ± 0.56	3.46 ± 0.65	.528
Hct (L/L)	0.32 ± 0.05	0.32 ± 0.04	0.32 ± 0.06	.709
White blood cell (×10^9^/L)	6.06 ± 2.12	5.97 ± 1.89	6.19 ± 2.43	.674
Neutrophils (×10^9^/L)	4.17 ± 1.99	4.02 ± 1.57	4.39 ± 2.47	.463
Platelet (×10^9^/L)	172.03 ± 56.62	171.87 ± 59.1	172.25 ± 54.04	.979
Lymphocyte (×10^9^/L)	1.19 ± 0.42	1.21 ± 0.42	1.17 ± 0.41	.747
Dosage of roxadustat (mg)	300 (210, 300)	300 (150, 300)	300 (270, 360)	.139
Plasma concentration (ng/mL)	521.85 ± 728.71	326.19 ± 345.5	794.39 ± 998.85	.008
Ferritin (ng/mL)	102.29 ± 154.63	72.64 ± 73.42	142.92 ± 217.96	.072
PTH (pg/mL)	361.47 ± 463.98	422.01 ± 552.06	276.25 ± 288.64	.215
Baseline Hb (g/L)	103.24 ± 17.66	109.51 ± 14.7	94.5 ± 17.95	<.001
Oral iron	46%	41%	54%	.31
Hb after 4 weeks (g/L)	103.64 ± 14.35	102.08 ± 14.38	105.5 ± 14.46	.438
Hb after 8 weeks (g/L)	107.26 ± 15.65	102.24 ± 16.68	112.29 ± 13.16	.06

BMI: body mass index; Hct: hematocrit; NLR: neutrophil-to-lymphocyte ratio; PLR: platelet-to-lymphocyte ratio; PTH: parathyroid hormone; Hb: hemoglobin; rhEPO: recombinant human erythropoietin; *spKt*/*V*: single pool *Kt/V*.

Previous treatment: previous anemia treatment from 3 to 6 months before enrollment.

### Independent influencing factors of clinical efficacy of roxadustat

The differences in the general characteristics between the response and non-response groups are listed in Table 1. Hb was 103.64 g/L after four weeks, and 107.26 g/L after 8 week. A total of 29 (43%) patients were included in the response group and 38 (57%) in the nonresponse group. There were no significant differences in terms of sex, weight, height, BMI, duration of dialysis, cause of kidney disease, previous treatment, RBC count, Hct, white blood cell count, PLT, dosage of roxadustat, ferritin, and PTH between the two groups. What stands out in the table is that blood trough concentration of roxadustat was higher in the response group (794.39 ± 998.85 vs. 326.19 ± 345.5, *p* = .008). The serum baseline Hb levels were significantly lower in the response group (*p* < .001), and the age was higher in patients who responded to roxadustat (58 ± 14.01 vs. 50.49 ± 11.43, *p* = .019).

**Figure 2.**
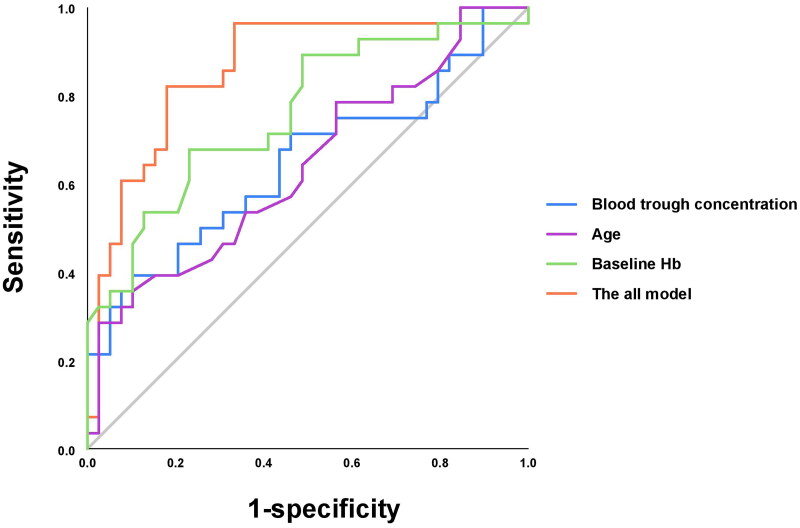
Receiver operating characteristic curve analysis evaluating the predictive ability of variables. The AUC of the model for predictive power of clinical efficacy was 0.859 (95%CI: 0.763–0.955); the AUC of blood trough concentration for predictive power of clinical efficacy was 0.647 (95%CI: 0.508–0.787); the AUC of age for predictive power of clinical efficacy was 0.64 (95%CI: 0.504–0.776); the AUC of baseline Hb for predictive power of clinical efficacy was 0.76 (95%CI: 0.642–0.897). AUC: area under the curve; Hb: hemoglobin.

Multivariable logistic regression showed that older age, higher blood trough concentration of roxadustat, and lower baseline Hb level were independent influencing factors of the response to roxadustat in hemodialysis patients (OR = 1.06, *p* = .025 for age; OR = 1.001, *p* = .037 for plasma concentration; and OR = 0.941, *p* = .003 for baseline Hb) (Table 2). In the ROC analysis, the AUC score was 0.859, with a sensitivity of 82% and specificity of 82% (Hosmer–Lemeshow’s test, *p* = .322) (Figure 2; Table 2).

**Table 2.** Univariate and multivariate analyses of influencing factors for clinical efficacy of roxadustat.

	Univariate analyses			Multivariate analyses		
	*β*	OR (95% CI)	*p*	*β*	OR (95% CI)	*p*
Age (yr)	0.047	1.049 (1.006, 1.092)	0.023	0.058	1.06 (1.007, 1.007)	0.025
Plasma concentration (ng/ml)	0.001	1.001 (1, 1.002)	0.015	0.001	1.001 (1.000, 1.003)	0.037
Baseline Hb (g/L)	−0.058	0.944 (0.911, 0.978)	0.001	−0.061	0.941 (0.904, 0.904)	0.003
Ferritin (ng/ml)	0.003	1.003 (0.999, 1.008)	0.111			
Cause of kidney disease (%)	0.12	1.127 (0.806, 1.577)	0.484			
Dosage of roxadustat (mg)	0.005	1.005 (0.999, 1.011)	0.121			

Furthermore, ROC analyses showed a low predictive power for each of the above variables in predicting the response to roxadustat (AUC score of 0.647 for blood trough concentration of roxadustat; 0.64 for age; 0.76 for baseline Hb; all *p* < .05) (Figure 2).

## Discussion

In this observational study, we found that age, blood trough concentration of roxadustat, and baseline Hb level were independent predictors of the response to roxadustat in hemodialysis patients. The results demonstrated that older patients, patients with high blood trough concentrations of roxadustat, and patients with lower baseline Hb levels were more likely to respond to roxadustat.

Roxadustat (FG-4592), as a new oral medication to treat renal anemia, is a prolyl hydroxylase inhibitor that increases HIF transcriptional activity by stabilizing HIF-α subunits. Increased transcriptional activity can promote erythropoiesis by increasing endogenous EPO [17]. Multiple trials have demonstrated that roxadustat is no less effective than ESAs in patients undergoing hemodialysis [11]. Although extensive research has been carried out on the effectiveness of roxadustat for renal anemia [18,19], there are little published data on the concentration–effect relationship of roxadustat. Our study demonstrated that high blood trough concentration of roxadustat was an independent predictor of response to roxadustat in hemodialysis patients, which showed that the higher the blood trough concentration, the better was the clinical efficacy. Zhang et al. [20] revealed that the blood concentration of roxadustat was correlated with the clinical efficacy in patients undergoing maintenance hemodialysis, which was consistent with our study. The study included patients who were exposed to roxadustat for the first time, and the response was defined as a change in Hb from baseline of ≥10 g/L, which was different from our study. However, a simple comparative analysis was performed between the non-response and response groups, and a small sample size (46 patients) was included in the study.

Our data showed no significant correlation between the dosage of roxadustat and clinical efficacy. We considered the following two factors: (A) the impact of genetic polymorphism. In the liver, roxadustat undergoes phase I oxidation by CYP enzyme 2C8, and phase II glucuronidation by UGT1A9. The hypothesis was confirmed by Chen et al. [21], who found that UGT1A9 gene polymorphism significantly affected efficacy and trough concentration of roxadustat, and the anemic efficacy of roxadustat was related to trough concentration of drug. In this study, 22 CKD patients not receiving dialysis and complicated with renal anemia were enrolled. The results showed that elevated Hb values and roxadustat concentrations in UGT1A9 rs2070959 locus AA group were significantly lower than that in AG group. However, there was no significant difference in roxadustat dosage between response and nonresponse group, which was consistent with our research. (B) Small sample size. The study included a total of 67 patients which may have statistical errors. Our results showed that age was positively associated with clinical efficacy, indicating that older patients were more likely to respond to roxadustat. This may be related to the reduced clearance of roxadustat in older patients [15]. Komatsu et al. found that the roxadustat PK was affected by the phosphate binder (PB) on roxadustat bioavailability and age on the clearance of roxadustat [22]. Among patients aged ≥65 years, the roxadustat clearance was estimated to decrease by 20.8%.

A significant correlation was observed between the baseline Hb levels and clinical efficacy. Our study showed that patients with lower baseline Hb levels were more likely to respond to roxadustat. This may be attributed to the higher roxadustat dosage in the patients with lower baseline Hb levels (Supplemental Table 1).

Iron is a major component of Hb and is involved in erythrocyte differentiation and proliferation. Ensuring favorable iron metabolism is essential for the treatment of anemia [23,24]. Roxadustat can affect iron metabolism by increasing transferrin levels, enhancing the total iron-binding capacity, and stabilizing serum iron levels. These effects improve iron supply for hematopoiesis [25]. In our study, the SF level and the percentage of patients receiving oral iron therapy was higher in the response group than in the nonresponse group, without a significant difference. However, no significant correlation was found between these variables and clinical efficacy, consistent with a previous study [20]. What should be noted was that, even though without a significant difference, the levels of ferritin in the nonresponse group were two times lower than in the response group, which may contribute to the poor response to roxadustat, especially if patients in non-response group also had functional iron deficiency. Functional iron deficiency was defined by adequate iron stores but insufficient iron availability for incorporation into erythroid precursors, characterized by TSAT ≤20% and elevated ferritin levels. Unfortunately, the data on change in transferrin saturation (TSAT) and ferritin were not available in our study.

No significant relationship was found between NLR and PLR as inflammatory markers derived from complete blood count (CBC), and clinical efficacy in this study. Most hemodialysis patients are in a state of low-grade inflammation. Numerous studies have shown that inflammation is one of the most frequent factors in ESA hyporesponsiveness in patients [26–28]. However, in phase 3 studies from China and Japan, in patients with high CRP levels, no increase in roxadustat dose was required to maintain the target Hb, while higher doses of ESAs were required to maintain the target Hb [11, 29]. These results suggest that the erythropoietic effect of roxadustat is less affected by inflammation. This mechanism may be associated with the inhibition of hepcidin expression in patients with CKD. Elevated hepcidin levels can limit iron availability and impair its absorption. Hepcidin promotes ferroportin degradation and prevents the release of stored iron from enterocytes, macrophages, and hepatocytes, resulting in iron utilization disorders [30,31]. Hepcidin can also suppress iron absorption in the duodenum by downregulating apical DMT1 expression in enterocytes [32]. However, the CRP levels were not available in this study.

This study has some limitations. First, this was a single-center observational study with a relatively small sample size, which might have led to unavoidable selection bias. Second, some potential confounding factors that might affect clinical efficacy, such as CRP, serum iron, and TSAT, were not measured in the study. Finally, the follow-up period was relatively short. Therefore, the effects of the blood trough concentration of roxadustat on the clinical efficacy should be considered preliminary. Further prospective, larger multicenter clinical trials with longer follow-up periods are required to confirm this finding.

## Conclusions

In conclusion, the present study showed that age, blood trough concentration of roxadustat, and baseline Hb level were independent influencing factors of response to roxadustat in hemodialysis patients. Patients with older age, higher blood trough concentration of roxadustat or lower baseline Hb level were more likely to respond to roxadustat.

## Ethical approval

This study was approved by the Ethics Committee of Changzhou First People’s Hospital (approval no. (2023) 041) in accordance with the Declaration of Helsinki.

## Consent form

Written informed consent for participation was obtained from each participant after full disclosure of the study aim. The researcher assured the participants that their information would be confidential.

## Supplementary Material

Supplemental Material

## Data Availability

The datasets used and analyzed during the study are available from the corresponding author upon reasonable request.
