# Supplementary material for: Influencing factors of clinical efficacy of roxadustat among hemodialysis patients
Source: Ren Fail. 2024 Feb 12;46(1):2308701. doi: 10.1080/0886022X.2024.2308701 (PMC10863536; doi:10.1080/0886022X.2024.2308701)
Supplement: Supplemental Material [file IRNF_A_2308701_SM6068.pdf]

Supplement Table 1 Dosage of roxadustat in the two groups

Hb	Dosage of roxadustat(mg)	P
Hb < 103 g/L	300 (300, 360)	0.005
Hb > 103 g/L	255 (150, 300)	

Hb: hemoglobin
